# Association Between Vascular NOTCH3 Aggregation and Disease Severity in a CADASIL Cohort – Implications for *NOTCH3* Variant‐Specific Disease Prediction

**DOI:** 10.1002/ana.27240

**Published:** 2025-04-23

**Authors:** Minne N. Cerfontaine, Gido Gravesteijn, Remco J. Hack, Kyra L. Dijkstra, Mar Rodríguez‐Girondo, Benno Gesierich, Marie‐Noëlle W. Witjes‐Ané, Remco van Doorn, Marco Duering, Julie W. Rutten, Saskia A. J. Lesnik Oberstein

**Affiliations:** ^1^ Department of Clinical Genetics Leiden University Medical Center Leiden The Netherlands; ^2^ Department of Pathology Leiden University Medical Center Leiden The Netherlands; ^3^ Department of Biomedical Data Sciences Leiden University Medical Center Leiden The Netherlands; ^4^ Medical Image Analysis Centre (MIAC) and Department of Biomedical Engineering University of Basel Basel Switzerland; ^5^ Department of Geriatrics and Department of Psychiatry Leiden University Medical Center Leiden The Netherlands; ^6^ Department of Dermatology Leiden University Medical Center Leiden The Netherlands

## Abstract

**Objective:**

Vascular NOTCH3 protein ectodomain aggregation is a pathological hallmark of cerebral autosomal dominant arteriopathy with subcortical infarcts and leukoencephalopathy (CADASIL), a monogenic small vessel disease typically caused by cysteine‐altering variants in *NOTCH3*. Given their high population frequency, these *NOTCH3* variants are an important genetic contributor to stroke and vascular dementia worldwide. Disease severity in CADASIL is highly variable and is mainly determined by the position of the pathogenic *NOTCH3* variant in the NOTCH3 ectodomain. Here, we aimed to investigate the association between NOTCH3 aggregation load in skin vessels, cysteine‐altering *NOTCH3* variants, and disease severity in a prospective cohort study of 212 patients with CADASIL with 39 distinct cysteine‐altering *NOTCH3* variants.

**Methods:**

NOTCH3 aggregation load in skin vessels was determined by calculating the NOTCH3 score; the fraction of skin vessel wall area positive for NOTCH3 staining. Variant‐specific NOTCH3 scores were calculated for variants present in 10 or more participants, by averaging the NOTCH3 scores of individuals with that distinct variant. The associations between the NOTCH3 score, *NOTCH3* variants, and neuroimaging and clinical outcomes were investigated using multivariable linear mixed models, Cox regression, and mediation analyses.

**Results:**

The NOTCH3 score was significantly associated with lifetime stroke probability and small vessel disease neuroimaging outcomes, but not with age. Variant‐specific NOTCH3 scores reflected differences in disease severity associated with distinct *NOTCH3* variants.

**Interpretation:**

These findings suggest that differences in NOTCH3 aggregation propensity underlie the differences in disease severity associated with *NOTCH3* cysteine‐altering variants, and show that *NOTCH3*‐variant specific NOTCH3 scores can contribute to improved individualized disease prediction in CADASIL. ANN NEUROL 2025;98:273–285

Aggregation of the ectodomain of the NOTCH3 protein (NOTCH3^ECD^) in small‐ to medium‐sized brain arteries is a major driver of vascular pathology in cerebral autosomal dominant arteriopathy with subcortical infarcts and leukoencephalopathy (CADASIL).^1^ CADASIL is typically caused by cysteine‐altering missense variants in *NOTCH3*, which lead to aberrant disulfide bridge formation in one of the 34 epidermal growth factor‐like repeat (EGFr) domains of the NOTCH3^ECD^.^2^, ^3^, ^4^, ^5^ This causes NOTCH3^ECD^ multimerization and aggregation in the media of the vessel wall, with sequestration of extracellular matrix proteins, such as HTRA1, TIMP3, and vitronectin.^1^, ^6^, ^7^ CADASIL disease severity is highly variable,^8^, ^9^, ^10^, ^11^, ^12^ and cysteine‐altering *NOTCH3* variants (*NOTCH3* ^cys^) are an important contributor to both early‐ and late‐onset stroke and dementia worldwide.^8^, ^9^, ^10^, ^13^, ^14^

Vascular NOTCH3^ECD^ aggregation can be visualized as granular NOTCH3^ECD^‐positive staining using immunohistochemistry, or as granular osmiophilic material (GOM) using electron microscopy.^15^, ^16^ Although clinical signs and symptoms of CADASIL are confined to the brain, NOTCH3^ECD^ aggregation is present in small arteries in all organs, including the skin. NOTCH3^ECD^ aggregation load in skin vessels has been shown to correlate with NOTCH3^ECD^ aggregation load in brain vessels.^16^

Hallmark symptoms in patients with CADASIL include mid‐adult onset of ischemic strokes and cognitive decline, neuropsychiatric disturbances such as apathy and depression, and migraine with aura. Brain magnetic resonance imaging (MRI) features are white matter hyperintensities (WMHs), lacunes, perivascular spaces (PVSs), cerebral microbleeds (CMBs), and brain atrophy.^17^ CADASIL disease severity is strongly associated with the position of the *NOTCH3* variant along one of 34 EGFr domains of the NOTCH3 protein.^18^, ^19^, ^20^, ^21^, ^22^, ^23^, ^24^, ^25^ *NOTCH3* ^cys^ are stratified into 3 risk categories for developing severe disease, based on odds ratios of patient‐to‐population frequencies of the EGFr domain in which they are located: low‐risk (LR‐*NOTCH3*), moderate‐risk (MR‐*NOTCH3*), or high‐risk (HR‐*NOTCH3*) variants.^20^

Recently, in small sample sizes, HR‐*NOTCH3* variants have been shown to be associated with a higher NOTCH3^ECD^ aggregation load than MR‐ and LR‐*NOTCH3* variants,^16^, ^20^ suggesting a relation between vascular NOTCH3^ECD^ aggregation load and disease severity. A direct association between NOTCH3^ECD^ aggregation load and disease severity has never been studied. Understanding the interplay between *NOTCH3* variants, NOTCH3^ECD^ aggregation, and disease severity is particularly relevant in the light of the current development of disease modifying therapies targeting vascular NOTCH3 aggregates.^26^, ^27^ In this prospective study of 212 individuals with a *NOTCH3* ^cys^ variant from CADASIL pedigrees,^18^, ^28^ we investigated whether NOTCH3^ECD^ aggregation load in skin vasculature is associated with disease severity, and with distinct *NOTCH3* ^cys^ variants.

## Methods

### Study Participants

Participants of the prospective CADASIL cohort Disease Variability in *NOTCH3*‐associated Small Vessel Disease (DiViNAS) were included, consisting of patients with CADASIL and premanifest relatives with a *NOTCH3* ^cys^ variant. A table of the 39 distinct *NOTCH3* variants in DiViNAS participants can be found in Supplementary Table S1. Details concerning participant inclusion and the study protocol have been published elsewhere.^18^, ^28^ The study was approved by the medical ethics committee Leiden‐The Hague‐Delft (P21.013, P18.164, and P17.170). All participants gave written informed consent and procedures were carried out in accordance with the Declaration of Helsinki.

All data from study participants included in DiViNAS was collected during 1 or 2 study visits (at baseline and at a follow‐up visit 2 years later). In addition to brain MRI, medical history, and neuropsychological testing, a 4‐mm skin punch biopsy was taken from the posterior upper arm. In addition, brain tissue of 12 deceased patients with CADASIL was collected. This paper follows the Strengthening the Reporting of Observational studies in Epidemiology (STROBE) guidelines.^29^

### Skin Biopsies and NOTCH3^ECD^ Immunohistochemistry

A total of 344 skin biopsies were taken of 218 study participants. Twenty‐two skin biopsies did not fulfill the criterion of having at least 20 blood vessels per slide. Two hundred twelve participants (HR‐*NOTCH3* n = 117, MR‐*NOTCH3* n = 88, LR‐*NOTCH3* n = 7) with at least 1 skin biopsy were included in the study, of which 110 also had a second skin biopsy taken at the 2‐year follow‐up time point. Skin biopsies were formalin fixed and embedded in paraffin. Per tissue specimen, two 5‐μm sections with a slice interval of 25 μm were pretreated with 0.1% trypsin for 30 minutes at 37°C and washed 3 times with phosphate‐buffered saline (PBS). The slides were then incubated for 2 hours at room temperature with a primary mouse anti‐NOTCH3^ECD^ antibody (clone 1E4, Millipore, dilution 1:1000, RRID:AB_2890101). A 2‐step detection system (Brightvision, ImmunoLogic, VWRKC‐DPVB55HRP) was used per the manufacturer's protocol. In short, the slides were post‐blocked for 15 minutes, washed with PBS, incubated for 30 minutes with the ready‐to‐use goat anti‐mouse/rabbit HRP antibody, washed in PBS, and subsequently stained with 3,3′‐Diaminobenzidine (DAB) + Substrate Chromogen System (Dako, K3468, diluted 1:50). Counterstaining was then performed with 1:10 diluted Harris Hematoxylin for 10 seconds. The slides were stained in 5 batches and the samples were randomly distributed to minimize batch effects. For each patient, baseline and follow‐up sections were stained in the same batch. In addition to skin, brain tissue of deceased patients with CADASIL with HR‐ (n = 9) and MR‐*NOTCH3* (n = 3) variants was sequentially sectioned with an interval of 5 μm and stained for NOTCH3^ECD^ according to the same protocol. Characteristics of the brain donors can be found in the Supplementary Table S2. The slides were scanned using the PANNORAMIC 250 slide scanner (3DHISTECH Kft, Budapest, Hungary) with 52 × magnification and the full focus setting in order to capture all NOTCH3^ECD^ granules within the 5‐μm thick slide.

### Quantification of NOTCH3^ECD^ Aggregation

**FIGURE 1:**
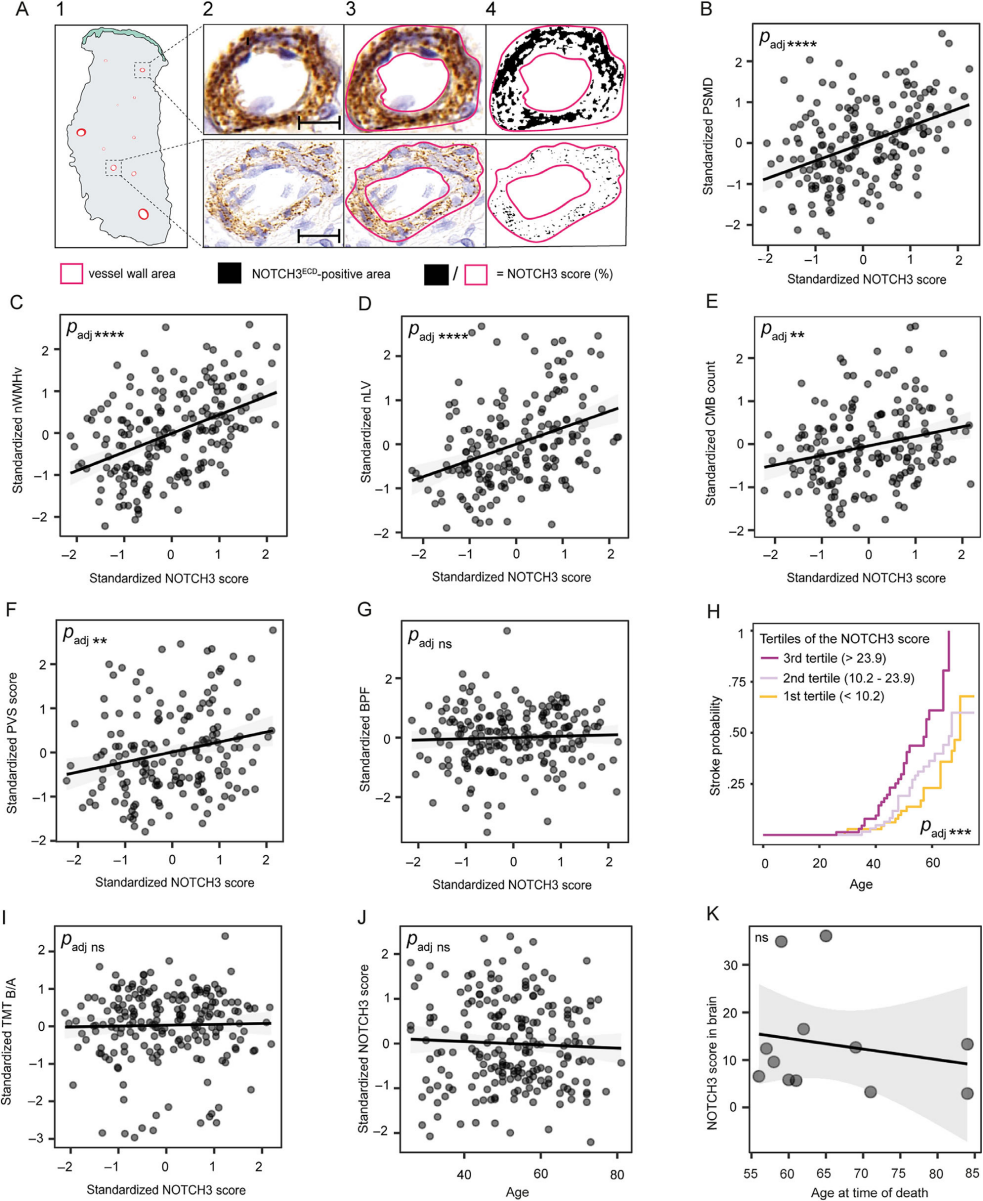
The association between the NOTCH3 score in skin vasculature with clinical and neuroimaging outcomes and with age. (A) Examples of NOTCH3 score measurements in two blood vessels. (1) Schematic overview of a skin biopsy, with the dermal and subcutaneous layers in gray and the epidermal layer in green. (2) Original images of blood vessels stained with a NOTCH3^ECD^‐antibody (*upper panel* scale bar = 10 μm and *lower panel* scale bar = 20 μm). (3) Region of interest drawn in pink (the blood vessel wall). (4) Thresholding of NOTCH3^ECD^ positive area. The NOTCH3 score was calculated by dividing the NOTCH3^ECD^‐positive area by the total vessel area, and then taking the average for the 10 most highest scoring vessels per sample. (B–G) Scatterplots with the standardized NOTCH3 score on the *x*‐axis (after square root transformation) and standardized neuroimaging outcomes on the *y*‐axis (conditional Pearson's residuals derived from linear mixed effects models corrected for age, sex, cardiovascular risk factors, and batch effects). There was a significant correlation between the NOTCH3 score and PSMD (*p* = 6.0 × 10^−11^, *p*_adj_ = 4.2 × 10^−10^ [B]), nWMHv (*p* = 5.4 × 10^−12^, *p*_adj_ = 4.3 × 10^−11^ [C]), nLV (*p* = 1.5 × 10^−7^, *p*_adj_ = 8.8 × 10^−7^ [D]), CMB count (*p* = 0.0017, *p*_adj_ = 0.0057 [E]), and PVS score (*p* = 0.0014, *p*_adj_ = 0.0057 [F]) but not with BPF (*p* = 0.72, *p*_adj_ = 1 [G]) after correction for age, sex, and cardiovascular risk factors and batch effects. (H) Kaplan–Meier plot showing the differences in stroke probability between the tertiles of the NOTCH3 score (with the first tertile containing those with the lowest score, and the third tertile the highest). A higher NOTCH3 score was associated with a higher lifetime stroke probability (*p* = 3.1 × 10^−5^, *p*_adj_ = 1.5 × 10^−4^ [H]), after correction for sex and cardiovascular risk factors and batch effects. There was no correlation between the NOTCH3 score and TMT_B/A_ (*p* = 0.74, *p*_adj_ = 1 [I]). (J–K) There was no association between age (range = 26–81 years) and the NOTCH3 score (*p* = 0.75, *p*_adj_ = 1 [J]). The NOTCH3 score of brain vessels was not correlated with the age of death (*p* = 0.54 [K]). PSMD = peak width of the skeletonized mean diffusivity; nWMHv = normalized white matter hyperintensity volume; nLV = normalized lacune volume; CMB = cerebral microbleed; PVS = perivascular space; BPF = brain parenchymal fraction; NOTCH3^ECD^ = NOTCH3 ectodomain; TMT_B/A_ = Trail Making Test B Given A *t* scores; *p*_adj_ = multiple correction adjusted *p* value (using the Holm method, n = 8 tested hypotheses for neuroimaging and clinical outcomes; n = 7 tested hypotheses for modifiers of the NOTCH3 score). Levels of significance: **** = *p* < 0.0001, *** = *p* < 0.001, ** = *p* < 0.01, * = *p* < 0.05, ns = not significant.

Quantification of NOTCH3^ECD^ aggregation (the NOTCH3 score) was performed as previously described.^16^ Briefly, vessel wall regions of interest were manually determined by 2 blinded observers (authors M.C. and G.G.) using QuPath,^30^ and the NOTCH3‐positive area was determined using Color Threshold in ImageJ (https://imagej.net/ij/) parameterized to capture NOTCH3 granules (hue 40–210 [stop], saturation 0–255, and brightness 0–150; Fig 1A). Next, the NOTCH3‐positive area was divided by the total vessel area, and the average of the 10 vessels with the highest score was calculated per sample. For each study participant, baseline and follow‐up samples were scored by the same observer. The inter‐rater reliability for the NOTCH3 score was determined in 60 random samples rated by 2 observers blinded to each other's scores, and to the genotype and clinical information of participants (single fixed rater intraclass correlation coefficient: 0.95, *p* < 2.2 × 10^−16^). Additionally, a variant‐specific NOTCH3 score (the average NOTCH3 score of participants with a distinct variant) was calculated for *NOTCH3* variants present in n ≥ 10 participants.

### Clinical and Neuroimaging Measures

Clinical outcomes were stroke, defined as neurological deficit lasting >24 hours in the absence of other probable causes or a diagnosis of ischemic stroke in the medical history, and executive function assessed by the Trail Making Test (TMT) A and B. Scores of the TMT B were corrected for age, sex, educational level, and TMT A‐score (TMT part B given A *t* scores, TMT_B/A_) using normative data reported from literature.^31^ Participants who were unable to finish the TMT part A or B in time (< 300 seconds) were scored as the lowest *t* score in the cohort, which was equal to 10. The following cardiovascular risk factors (CVRFs) were recorded: hypertension, hypercholesterolemia, diabetes type 1 or 2, and pack years of smoking, as defined for the DiViNAS cohort elsewhere.^18^, ^28^

Brain MRI was performed on a single 3T MRI‐system (Philips Diamond Select Achieva 3.0 [TX], Philips Medical Systems, Best, The Netherlands) using a 32‐channel head coil, and included the following sequences: 3D T1‐weighted images, T2‐weighted images, fluid‐attenuated inversion recovery, susceptibility‐weighted images, and diffusion‐weighted imaging with 30 directions. Detailed acquisition parameters and quantification methods are published elsewhere.^18^, ^28^ Small vessel disease (SVD) neuroimaging outcomes were peak width of the skeletonized mean diffusivity (PSMD), WMH volume (WMHv), brain volume, CMB count, lacune volume (LV), and a semiquantitative scale for PVS burden.^32^ WMHv, LV, and brain volumes were assessed following STRIVE‐2 consensus criteria^33^ and divided by the intracranial volume (ICV) to calculate normalized WMHv (nWMHv), normalized LV (nLV), and brain parenchymal fraction (BPF). PSMD was calculated from pre‐processed data using a publicly available script (https://github.com/miac‐research/psmd, version 1.8.3) with standard parameters.^34^

### Statistical Analysis

Statistical analysis was performed in R version 4.4.0. Variables with a normal distribution were reported using the mean ± standard deviation (SD); variables with a skewed distribution were reported using the median ± interquartile range (IQR). Multivariable linear mixed effects regression analyses were performed to investigate the association among PSMD (n = 192), nWMHv (n = 208), BPF (n = 209), nLV (n = 189), CMB count (n = 188), PVS score (n = 175), and TMT_B/A_ (n = 211) as dependent variables and the NOTCH3 score as independent variable using the *lme4* package. The PVS score was treated as a continuous variable to facilitate comparisons with other continuous variables, but a sensitivity analysis was performed with (quartiles of) the PVS score as an ordinal outcome. The association between the NOTCH3 score and lifetime stroke probability (n = 212) was assessed with multivariable Cox regression analysis using the *coxph* package. In all multivariable analyses, age (excluded in Cox regression), sex, and CVRFs were included as covariates next to the NOTCH3 score, with batch numbers included either as a random effect or as a cofactor for the Cox regression models. For the outcomes that were significantly associated with the NOTCH3 score, an additional multivariable analysis was performed including *NOTCH3* variant risk category.

To investigate modifiers of the NOTCH3 score, multivariable linear mixed effects regression analysis was performed with the following independent variables: *NOTCH3* variant risk category, age, sex, CVRFs, with batch numbers as a random effect. All analyses were performed for the neuroimaging data collected at baseline (the main dataset) and were confirmed independently for the data collected at 2‐year follow‐up (the replication dataset), except for the PVS score, which was only available at baseline. To compare the NOTCH3 score in brain vessels of patients with HR‐ and MR‐*NOTCH3* variants, a Wilcoxon signed rank test was performed; for testing the association between age at the time of death and the NOTCH3 score, the Spearman's rank correlation was used. To investigate whether there was a significant 2‐year change in the NOTCH3 score (n = 110; Supplementary Table S1), a linear mixed model (LMM) was created with follow‐up time as a continuous fixed effect with crossed random intercepts for ID and batch number, and compared with a null model using a likelihood‐ratio test.

Mediation analyses were performed between *NOTCH3* variant risk category, the NOTCH3 score and clinical and neuroimaging outcomes. This was only performed for outcomes that were significantly associated with the NOTCH3 score in a multivariable model after stratification for *NOTCH3* variant risk category. In each model, we included age, sex, and CVRFs as fixed effects, and batch numbers as a random effect. CVRFs, age, and sex were included as confounders as these have been consistently reported to be key modifiers of CADASIL disease severity.^13^, ^18^, ^23^, ^35^ A Quasi‐Bayesian Confidence Intervals approach with 10,000 simulations was performed using the *mediation* R package. Sensitivity analyses on the mediation effects for violations of the assumption of no unmeasured confounding were conducted using the *medsens* function in the *mediation* package. Because this function does not support LMMs, mediation analyses were also performed using linear regression models; plots of the average mediation effects as a function of the sensitivity parameter rho^36^ are shown in Supplementary Figure S3.

For the analyses of the NOTCH3 score of distinct *NOTCH3* variants, the main and validation datasets were combined. To examine to what extent intervariant differences explained the additional variability in the NOTCH3 score, a likelihood‐ratio test was performed between an LMM of the NOTCH3 score that only included *NOTCH3* variant risk category, and an LMM with each EGFr domain as an additional cofactor in all patients with HR‐ and MR‐*NOTCH3* variants. The variant‐specific NOTCH3 scores were only calculated for *NOTCH3* variants present in 10 participants to ensure reliable estimates of averages, and were equal to the estimated marginal means (EMMs) per variant derived from an LMM with ID and batches as crossed random effects, and distinct *NOTCH3* variant and sex as fixed effects, using the *emmeans* package. Each participant with a certain *NOTCH3* variant was then assigned this variant‐specific NOTCH3 score. For those outcomes that were significantly associated with the NOTCH3 score, LMMs and Cox regression analyses were performed to assess the association between the variant‐specific NOTCH3 score and disease severity. Comparisons were performed between models to predict disease severity using the variant‐specific NOTCH3 score and a participant's individual NOTCH3 score using Akaike Information Criteria (AIC). Differences in AICs of more than 2 were considered evidence that the lowest scoring model performed better.

To obtain plausible normal or homoscedastic residual distribution for linear mixed effects regression analyses, transformations were performed for PSMD and CMB count (natural logarithm), nWMHv (square root), and nLV (cube root). For all analyses, the NOTCH3 score was square root transformed and pack years were natural logarithm transformed. All continuous dependent and independent variables were standardized and scaled. Two‐sided *p* values < 0.05 were considered statistically significant. Correction for multiple testing for neuroimaging and clinical outcomes and modifiers of the NOTCH3 score was performed using the Holm method, and reported as adjusted *p* value (*p*_adj_).

## Results

### The Association Between the NOTCH3 Score and Neuroimaging and Clinical Outcomes

Descriptive characteristics of study participants are summarized in the Table 1. The NOTCH3 score (Fig 1B‐G) was significantly associated with PSMD (*β* = 0.34, 95% confidence interval [CI] = 0.24 to 0.43, *p* = 6.0 × 10^−11^, *p*_adj_ = 4.2 × 10^−10^), nWMHv (*β* = 0.38, 95% CI = 0.28 to 0.48, *p* = 5.4 × 10^−12^, *p*_adj_ = 4.3 × 10^−11^), nLV (*β* = 0.34, 95% CI = 0.22 to 0.46, *p* = 1.5 × 10^−7^, *p*_adj_ = 8.8 × 10^−7^), CMB count (*β* = 0.20, 95% CI = 0.08 to 0.32, *p* = 0.0017, *p*_adj_ = 0.0057), and PVS score (*β* = 0.22, 95% CI = 0.09 to 0.36, *p* = 0.0014, *p*_adj_ = 0.0057), but not with BPF (*β* = 0.017, 95% CI = −0.076 to 0.11, *p* = 0.72, *p*_adj_ = 1). The statistical inference of the NOTCH3 score remained significant when considering PVS score as an ordinal outcome (Supplemental Results; Data S1).

**TABLE 1:** Descriptive Characteristics

Number of Participants, n	212
*NOTCH3* variant risk category, n (%)	
HR*‐NOTCH3*	117 (55.2)
MR*‐NOTCH3*	88 (41.5)
LR*‐NOTCH3*	7 (3.3)
Age, yr, mean (SD)	52.3 (12.2)
Male sex, n (%)	101 (47.6)
NOTCH3 score, median (range, IQR)	15.7 (0.86–55.9, 21.1)
Ischemic stroke, n (%)	71 (33.5)
Age of first stroke, yr, mean (SD)	50.9 (11.6)
Hypertension, n (%)	50 (23.6)
Hypercholesterolemia, n (%)	80 (37.8)
Diabetes type 1 or 2, n (%)	12 (5.7)
Pack years, median (IQR)	0.25 (10.1)

HR*‐NOTCH3* = high‐risk *NOTCH3* variant risk category; MR*‐NOTCH3* = moderate‐risk *NOTCH3* variant risk category; LR*‐NOTCH3* = low‐risk *NOTCH3* variant risk category; IQR = interquartile range.

The NOTCH3 score was significantly associated with a higher life‐time risk of stroke (hazard ratio = 1.8, 95% CI = 1.4 to 2.4, *p* = 3.1 × 10^−5^, *p*_adj_ = 1.5 × 10^−4^; Fig 1H). There was no association between the NOTCH3 score and TMT_B/A_ (*β* = 0.024, 95% CI = −0.13 to 0.16, *p* = 0.74, *p*_adj_ = 1; Fig 1I). There was no association between age (range = 26–81 years) and the NOTCH3 score in skin (*β* = 0.021, 95% CI = −0.10 to 0.15, *p* = 0.75, *p*_adj_ = 1; Fig 1J) or brain vessels (*p* = 0.54; Fig 1K). There was no significant 2‐year change in the NOTCH3 score (*β* = −0.078, 95% CI = −0.21 to 0.057, *p* = 0.25).

### The Association between the NOTCH3 Score and *NOTCH3* Variant Risk Categories

**FIGURE 2:**
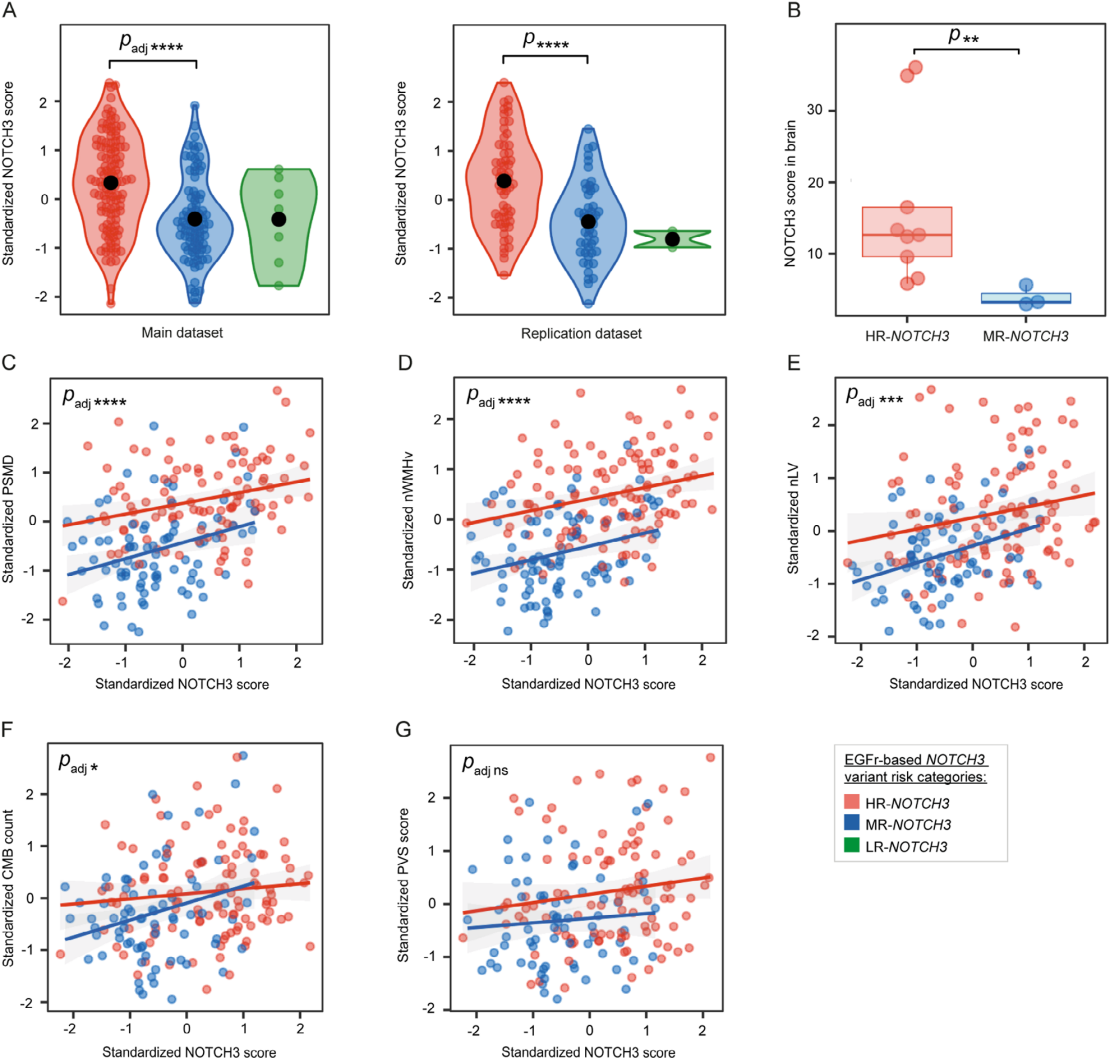
The association between the NOTCH3 score and *NOTCH3* variant risk categories. (A) Violin plots with *NOTCH3* variant risk group on the *x‐*axis and standardized skin NOTCH3 score per participant on the *y*‐axis. The standardized NOTCH3 score is equal to the conditional Pearson's residuals derived from linear mixed effects models corrected for sex, age, and CVRF and batch effects. There was a significant difference in the NOTCH3 score between participants with HR‐ and MR‐*NOTCH3* variants in the main dataset (*p* = 5.7 × 10^−9^, *p*_adj_ = 4.0 × 10^−8^) and in the replication dataset (*p* = 1.5 × 10^−6^). (B) In brain vessels, the NOTCH3 score in the HR‐*NOTCH3* group was significantly higher than in the MR‐*NOTCH3* group (*p* = 0.0091). (C–G) The association between the NOTCH3 score and PSMD (*β* = 0.22, 95% CI = 0.12 to 0.31, *p* = 1.6 × 10^−5^, *p*_adj_ = 9.5 × 10^−5^ [C]), nWMHv (*β* = 0.22, 95% CI = 0.12 to 0.32, *p* = 1.8 × 10^−5^, *p*_adj_ = 9.5 × 10^−5^ [D]), nLV (*β* = 0.24, 95% CI = 0.11 to 0.37, *p* = 6.0 × 10^−4^, *p*_adj_ = 0.0024 [E]), and CMB count (*β* = 0.17, 95% CI = 0.039 to 0.29, *p* = 0.013, *p*_adj_ = 0.039 [F]) remained significant after stratification for *NOTCH3* variant risk category, but not PVS score (*β* = 0.13, 95% CI = −0.015 to 0.28, *p* = 0.087, *p*_adj_ = 0.087 [G]). PSMD = peak width of the skeletonized mean diffusivity; nWMHv = normalized white matter hyperintensity volume; nLV = normalized lacune volume; CMB = cerebral microbleed; PVS = perivascular space; HR‐*NOTCH3* = high‐risk NOTCH3 variant risk category; MR‐*NOTCH3* = moderate‐risk NOTCH3 variant risk category; LR‐*NOTCH3* = low‐risk NOTCH3 variant risk category; *p*_adj_ = multiple correction adjusted *p* value (using the Holm method, n = 6 tested hypotheses for neuroimaging and clinical outcomes; n = 7 tested hypotheses for modifiers of the NOTCH3 score). Levels of significance: **** = *p* < 0.0001, *** = *p* < 0.001, ** = *p* < 0.01, * = *p* < 0.05, ns = not significant.

Participants with an HR‐*NOTCH3* variant had a significantly higher NOTCH3 score than participants with an MR‐*NOTCH3* variant (*β* = 0.78, 95% CI = 0.54 to 1.0, *p* = 5.7 × 10^−9^, *p*_adj_ = 4.0 × 10^−8^; Fig 2A). In brain vessels, the NOTCH3 score in the HR‐*NOTCH3* group was significantly higher than in the MR‐*NOTCH3* group (*p* = 0.0091; Fig 2B). The association between age and the NOTCH3 score did not differ significantly between the HR‐ and MR‐*NOTCH3* groups (*p* = 0.14; Supplementary Fig S1). The NOTCH3 score was found to be a significant mediator of neuroimaging outcomes and lifetime stroke probability (Supplementary Fig S2; for a sensitivity analysis, see Supplementary Fig S3).

After stratification for *NOTCH3* variant risk category, the association between the NOTCH3 score and neuroimaging outcomes and lifetime stroke probability remained significant, but not PVS score (Fig 2C–G). The associations between the NOTCH3 score and all neuroimaging markers were confirmed in the replication dataset, except for CMB count when stratifying for *NOTCH3* variant risk category (see Supplemental Results; Data S1). Male participants had a lower NOTCH3 score than female participants in the main dataset (*β* = −0.39, 95% CI = −0.63 to −0.15, *p* = 0.0019, *p*_adj_ = 0.011; see Supplementary Fig S1), but this was not confirmed in the replication dataset. There was no association between CVRF and the NOTCH3 score (*p*_adj_ = 1 for all CVRF).

### The NOTCH3 Score of Distinct *NOTCH3* Variants and the Association With Disease Severity Outcomes

**FIGURE 3:**
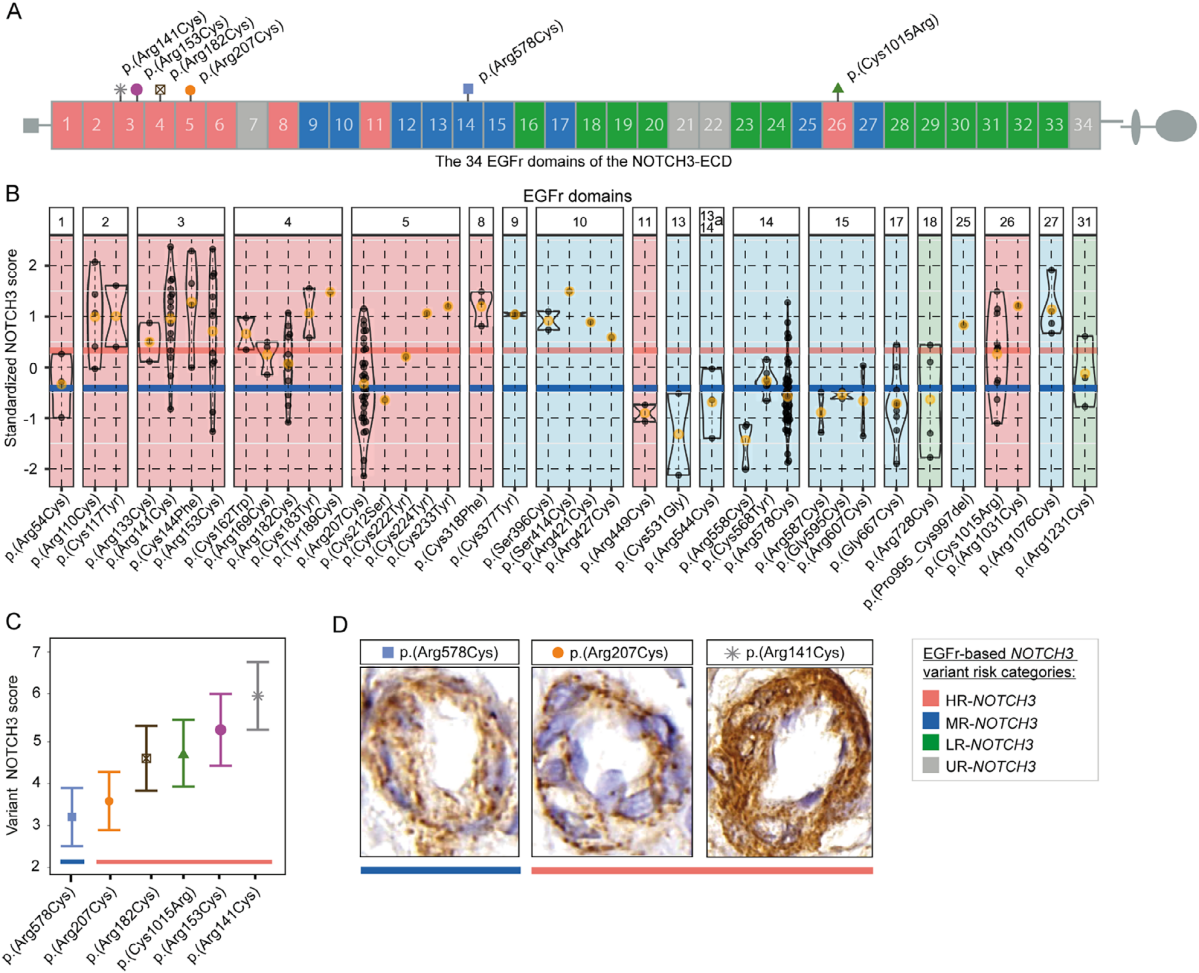
The association between the NOTCH3 score and distinct *NOTCH3* variants. (A) A schematic overview of the NOTCH3 protein. The ECD of the NOTCH3 protein includes 34 EGFr domains. Each EGFr domain contains numerous distinct *NOTCH3* variants that may lead to CADASIL, the majority of which are cysteine‐altering missense variants. Distinct *NOTCH3* variants which were represented by 10 or more participants are denoted by symbols above the EGFr domains in which they are located. *NOTCH3* variants are classified into *NOTCH3* variant risk categories based on the EGFr domain in which they are situated, and can be classified into high risk (HR‐*NOTCH3*, in *red*), moderate risk (MR‐N*OTCH3*, in *blue*), low risk (LR‐*NOTCH3*, in *green*) or unknown (UR‐*NOTCH3*, in *gray*) categories. These *NOTCH3* variant risk categories were published elsewere^20^ and are based on cohort‐to‐population frequency odds ratios of the EGFr domains these *NOTCH3* variants are located in. (B) Violin plots of the NOTCH3 score with on the x‐axis distinct *NOTCH3* variants grouped per EGFr domain (in *red* HR‐*NOTCH3* variants; in *blue* MR‐*NOTCH3* variants; and in *green*, LR‐*NOTCH3* variants) and on the *y*‐axis, the standardized NOTCH3 score (conditional Pearson's residuals derived from mixed effects models corrected for age, sex, CVRF, and batch effects). The black dots represent the NOTCH3 score for one individual participant; the orange dot represents the average for a single *NOTCH3* variant. The red line represents the average for all HR‐*NOTCH3* variants combined, whereas the blue line is the average for all MR‐*NOTCH3* variants combined. Differences between EGFr domains accounted for a significant proportion of variability observed in the NOTCH3 score in the HR‐ and MR‐*NOTCH3* groups (*p* = 1.0 × 10^−19^). Some of the *NOTCH3* variants had a striking deviation of the NOTCH3 score from their respective *NOTCH3* variant risk categories, including the HR‐*NOTCH3* variants p.(Arg207Cys; n = 33), p.(Arg449Cys; n = 2), and the MR‐*NOTCH*3 variants in EGFr domains 9 and 10 (n = 7), and p.(Arg1076Cys; n = 4). As some variants are overrepresented in EGFr domains, and due to power considerations, further analyses of disease severity were performed using only those distinct *NOTCH3* variants with 10 or more participants (C). (C) The variant‐specific NOTCH3 score (variant NOTCH3 score) with 95% confidence intervals, sorted in ascending order. The blue and the red lines indicate the *NOTCH3* variant risk category (*blue* = MR‐*NOTCH3*; *red* = HR‐*NOTCH3*). (D) Images depicting blood vessels with representative granular NOTCH3^ECD^ staining with a NOTCH3 score equal to that of their respective variant. The images of p.(Arg578Cys) and p.(Arg207Cys) variants show similar NOTCH3^ECD^ aggregation, whereas the p.(Arg141Cys) variant shows more NOTCH3^ECD^ aggregation. EGFr = epidermal growth factor‐like repeat; HR‐*NOTCH3* = high‐risk *NOTCH3* variant risk category; MR‐*NOTCH3* = moderate‐risk *NOTCH3* variant risk category; LR‐*NOTCH3* = low‐risk *NOTCH3* variant risk category; UR‐*NOTCH3* = unknown‐risk *NOTCH3* variant risk category. ^a^The p.(Arg544Cys) variant is located between EGFr domains 13 and 14.

**FIGURE 4:**
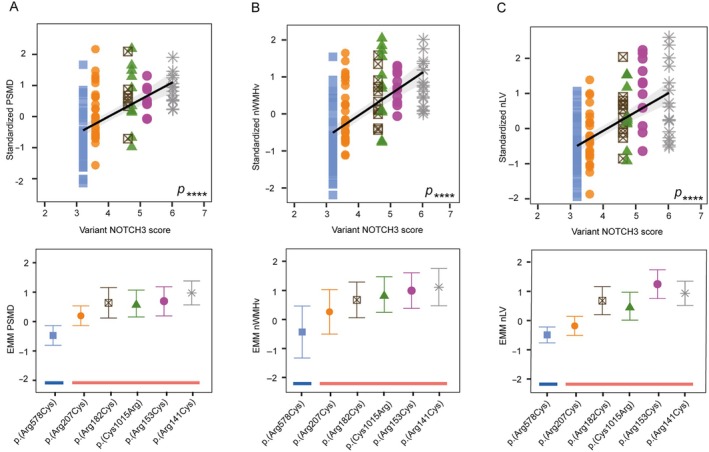
The association between variant‐specific NOTCH3 scores and disease severity. (A, B) Upper panels. The variant‐specific NOTCH3 score (variant NOTCH3 score) was significantly associated with PSMD (*p* = 6.3 × 10^−13^ [D]), nWMHv (*p* = 1.4 × 10^−17^ [E]), nLV (*p* = 4.2 × 10^−14^ [F]) and performed better than using an individual participant's NOTCH3 score in predicting disease severity (see Supplementary Table S4). (A, B) Lower panels. EMMs with 95% confidence intervals of PSMD (D), nWMHv (E), and nLV (F) per variant, arranged in ascending order of the variant‐specific NOTCH3 score. The blue and red lines underneath each datapoint represent the *NOTCH3* variant risk category (*red* = HR‐*NOTCH3*, *blue* = MR‐*NOTCH3*). EMM = estimated marginal means; PSMD = peak width of the skeletonized mean diffusivity; nWMHv = normalized white matter hyperintensity volume; nLV = normalized lacune volume. Levels of significance: **** = *p* < 0.0001.

The 39 distinct NOTCH3 variants of the DiViNAS participants were distributed over 19 EGFr domains (Fig 3 and Supplementary Table S1). Differences between EGFr domains accounted for a significant proportion of variability observed in the NOTCH3 score in the HR‐ and MR‐*NOTCH3* groups (*p* = 1.0 × 10^−19^). The average NOTCH3 score of several distinct *NOTCH3* variants deviated from the average of their *NOTCH3* variant risk category classification (see Fig 3). The most frequent *NOTCH3* variants (present in n ≥ 10 participants) were the MR‐*NOTCH3* variant p.(Arg578Cys; n = 47), and the HR‐*NOTCH3* variants p.(Arg141Cys; n = 16), p.(Arg153Cys; n = 11), p.(Arg182Cys; n = 12), p.(Arg207Cys; n = 33), and p.(Cys1015Arg; n = 10). The variant‐specific NOTCH3 scores of these 6 variants (Fig 3C, D) were significantly associated with PSMD (*β* = 0.50, 95% CI = 0.38 to 0.62, *p* = 6.3 × 10^−13^), nWMHv (*β* = 0.56, 95% CI = 0.45 to 0.67, *p* = 1.4 × 10^−17^), nLV (*β* = 0.59, 95% CI = 0.46 to 0.72, *p* = 4.2 × 10^−14^) (Fig 4A–C), CMB count (*β* = 0.30, 95% CI = 0.16 to 0.45, *p* = 6.7 × 10^−5^), PVS score (*β* = 0.30, 95% CI = 0.13 to 0.47, *p* = 5.6 × 10^−4^) and lifetime stroke probability (hazard ratio = 2.3, 95% CI = 1.6 to 3.2, *p* = 1.6 × 10^−6^). The variant‐specific NOTCH3 score performed better than an individual participant's NOTCH3 score in predicting disease severity outcomes (Supplementary Table S4).

**FIGURE 5:**
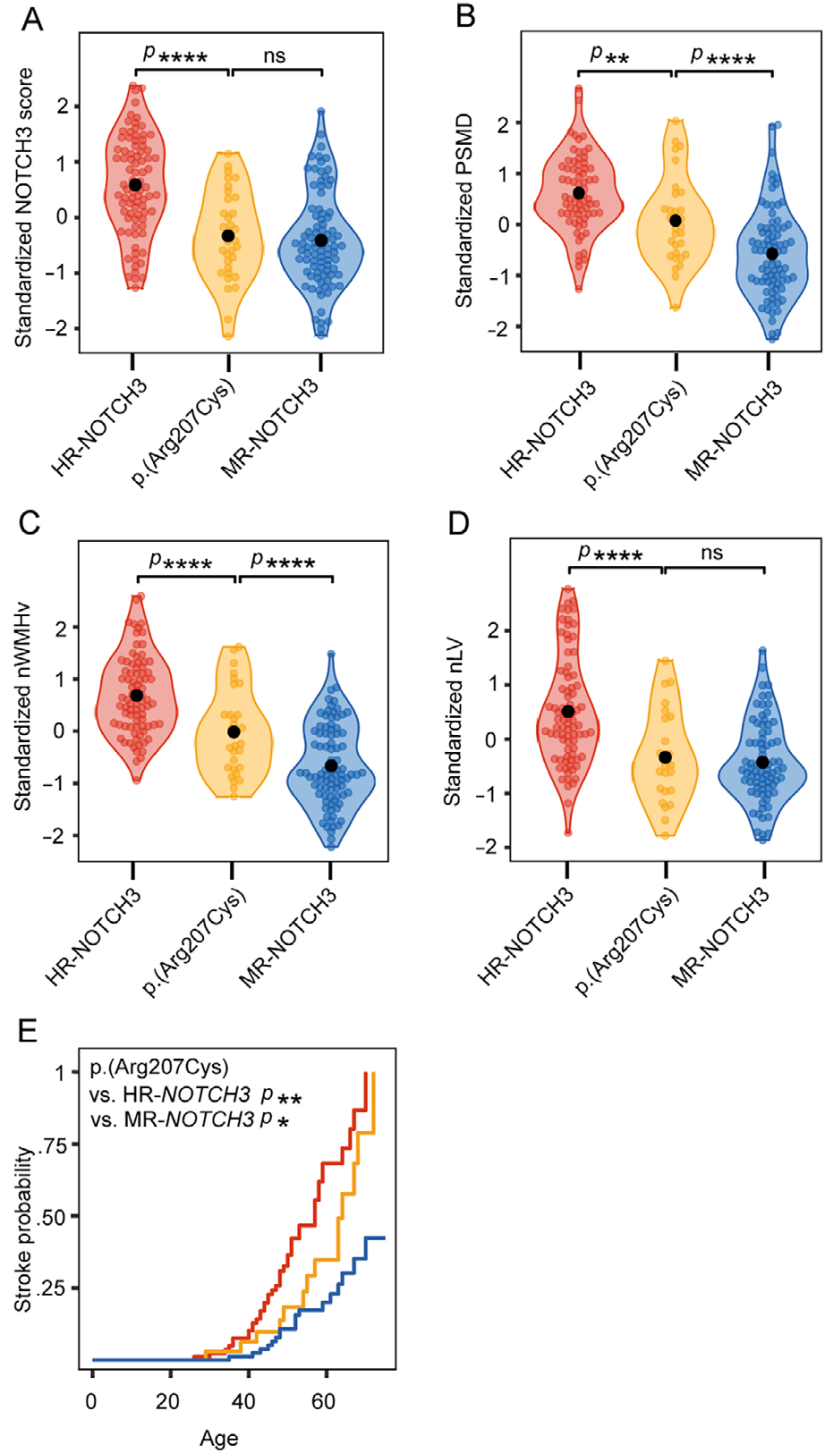
The NOTCH3 score and disease severity of the p.(Arg207Cys) variant. Using a variant‐specific NOTCH3 score‐first approach may provide a framework for increasing the resolution of clinically relevant risk classification on the level of distinct *NOTCH3* variants. The HR*‐NOTCH3* p.(Arg207Cys) variant was associated with a lower variant‐specific NOTCH3 score than the average of other HR‐*NOTCH3* variants (*p* = 4.9 × 10^−11^), and did not differ from the average NOTCH3 score of the MR‐*NOTCH3* group (*p* = 0.60 [A]). The p.(Arg207Cys) variant was significantly less severe than the average of the HR*‐NOTCH3* group for PSMD (*p* = 1.6 × 10^−3^ [B]), nWMHv (*p* = 4.8 × 10^−6^ [C]), nLV (*p* = 1.1 × 10^−6^ [D]), and lifetime stroke probability (*p* = 0.0061 [E]), and more severe than the MR‐*NOTCH3* group for PSMD (*p* = 7.2 × 10^−5^ [B]), nWMHv (*p* = 2.2 × 10^−5^ [C]), and lifetime stroke probability (*p* = 0.045 [E]). PSMD = peak width of the skeletonized mean diffusivity; nWMHv = normalized white matter hyperintensity volume; nLV = normalized lacune volume; HR‐*NOTCH3* = high‐risk *NOTCH3* variants; MR‐*NOTCH3* = moderate‐risk *NOTCH3* variants; Levels of significance: **** = *p* < 0.0001, ** = *p* < 0.01, * = *p* < 0.05, ns = not significant.

The variant‐specific NOTCH3 score of p.(Arg207Cys) was observed to be notably lower than that of other HR‐*NOTCH3* variants. This was statistically significant (*β* = 1.1, 95% CI = 0.78 to 1.4 *p* = 4.9 × 10^−11^), and the variant‐specific NOTCH3 score of the p.(Arg207Cys) variant was comparable to that of MR‐*NOTCH3* variants (*β* = −0.079, 95% CI = −0.37 to 0.21, *p* = 0.60) (Fig 5A). Participants with the p.(Arg207Cys) variant also had a significantly lower PSMD, nWMHv, nLV, and lifetime stroke probability than participants with other HR‐*NOTCH3* variants (Fig 5B–E); CMB count did not significantly differ (see Supplementary Table S5 for coefficients and *p* values). The average NOTCH3 score of the p.(Arg578Cys) variant did not differ significantly from the average of the other MR‐*NOTCH3* variants, although there was a trend toward a lower NOTCH3 score in the p.(Arg578Cys) variant (reference group = p.[Arg578Cys], *β* = 0.33, 95% CI = −0.0001 to 0.66, *p* = 0.055).

## Discussion

The findings of this study give novel insights into the interplay between cysteine‐altering *NOTCH3* variants, vascular NOTCH3^ECD^ protein aggregation, and *NOTCH3*‐associated small vessel disease severity. We establish that (i) NOTCH3^ECD^ aggregation load in skin vessels is associated with neuroimaging outcomes and stroke risk, and (ii) that the average NOTCH3 score of distinct *NOTCH3* variants is a predictor of disease severity, contributing to an improved genotype‐based disease prediction.

*NOTCH3* ^cys^ variants occur at an estimated population frequency of 1 in 300 and are associated with a highly variable small vessel disease severity, with CADASIL representing the severe end of the spectrum.^8^, ^9^, ^10^, ^11^, ^12^ We previously showed that *NOTCH3* variants can be classified into 3 risk categories for developing severe disease.^20^ In this study, we demonstrate that the differences in disease severity between individuals with HR‐ and MR‐*NOTCH3* variants is mediated through differences in NOTCH3^ECD^ aggregation load. However, even within these risk categories and when accounting for cardiovascular risk burden, there remains a significant unexplained variability in disease severity. The results show that the NOTCH3 score can be used to more accurately predict disease severity associated with a particular *NOTCH3* variant, paving the way for improved individualized genotype‐based disease prediction in the clinic.

Our data suggest that *NOTCH3* ^cys^ variants, of which over 370 have been reported,^20^ are associated with differential NOTCH3^ECD^ aggregation load at the individual variant level, although there seems to be some topographical clustering along the NOTCH3 ectodomain of variants with similar NOTCH3 scores. This could be due to the 3‐dimensional configuration of the NOTCH3 protein,^20^ or the proximity to non‐enzymatic NOTCH3 cleavage sites.^37^, ^38^ The amino acid that replaces, or is replaced by, a cysteine residue does not seem to be a major determinant of mutant protein properties.^39^ Other factors may include expression levels of NOTCH3 and of extracellular matrix proteins known to be sequestered in NOTCH3^ECD^ aggregates.^1^, ^40^, ^41^, ^42^ Vascular NOTCH3^ECD^ aggregation is likely a complex interplay of these and other factors within the specific context of the vessel wall.

We did not find an association between CVRF burden and NOTCH3^ECD^ aggregation, and an inconsistent association with male sex. These known modifiers of CADASIL severity^13^, ^18^, ^23^, ^35^ therefore likely do not exert their effect via the NOTCH3^ECD^ aggregation pathway. There was no association of the NOTCH3 score with BPF or with TMT_B/A_, in line with what was found previously for *NOTCH3* variant risk category.^18^ In vitro assays in patient‐derived inducible pluripotent stem cell 3D‐vessel on chip models,^43^ comparative multi‐omics studies in patient tissues,^41^ as well as advances in artificial intelligence (AI)‐driven protein 3D modeling tools, such as AlphaFold,^44^ may contribute to our further understanding of NOTCH3^ECD^ aggregation and its modifiers.

Given the fact that CADASIL is a progressive disorder and it has been shown that the cerebrovascular NOTCH3 score increases with age in CADASIL mouse models,^15^, ^26^ we had expected to find an association between the NOTCH3 score and age, which was not the case. We cannot rule out that technical limitations or a bias in our cohort precluded the capture of such an association. On the other hand, these findings may imply that NOTCH3^ECD^ aggregation as measured with the NOTCH3 score reaches a plateau somewhere in the first 2 decades of life, as NOTCH3^ECD^ aggregation in skin vessels has been shown to be present in individuals as young as 19 years of age.^16^, ^45^, ^46^, ^47^ This hypothesis is further corroborated by the fact that we found no association between the NOTCH3 score in brain vessels and age at the time of death, whereas there was a significant difference in the NOTCH3 score of brain vessels between patients with HR‐ versus MR‐*NOTCH3* variants. Although we have previously shown an association between the NOTCH3 score in skin and brain,^16^ progression of NOTCH3^ECD^ aggregation in brain vessels likely differs from that in skin. The link between NOTCH3^ECD^ aggregation load and disease severity is probably manifold stronger if it could be measured in brain vessels using in vivo molecular imaging techniques. Development of such tools to measure cerebrovascular progression of NOTCH3^ECD^ aggregation may help to determine the optimal age to initiate treatment with disease modifying therapies targeting vascular NOTCH3^ECD^ aggregation.^26^, ^27^

Both a strength and limitation of our study is that some *NOTCH3* variants are prevalent in The Netherlands and are therefore over‐represented in the DiViNAS cohort, such as the MR‐*NOTCH3* p.(Arg578Cys) and HR‐*NOTCH3* p.(Arg207Cys) variants. This provided enough statistical power to compare some distinct *NOTCH3* variants with one another, but these over‐represented variants prohibited the distinction between aggregation properties of distinct variants versus shared aggregation properties of variants within a specific EGFr domain. There were some variants which showed a striking deviation from the average NOTCH3 score of the *NOTCH3* variant risk category in which they are (currently) classified, such as the p.(Arg1076Cys) variant in EGFr domain 27. The sample size of this variant was too low to draw any conclusions regarding potential re‐classification of this variant, but the observation is intriguing, as EGFr 27 is located next to the HR‐*NOTCH3* EGFr domain 26. We could not study the NOTCH3 score in patients with non‐cysteine altering^5^, ^48^ or low‐risk *NOTCH3* variants, as these were not represented or were under‐represented in our study population. A sensitivity analysis of the mediation analyses showed that if there are unmeasured confounders, then these may strongly influence the estimated mediation effect of the NOTCH3 score. Although we cannot completely rule out such confounders, the results of the mediation analyses seem plausible, as we have included the main confounders previously reported in literature, and the mediation analyses showed consistent effects of the NOTCH3 score as a mediator for all associations between *NOTCH3* variant risk category and the 5 disease outcomes. Finally, it is important to stress that, in its current form, the NOTCH3 score of a single patient cannot be used for individualized disease prediction nor is it suitable as a biomarker for clinical trials, as the current technique is not sensitive enough due to experimental and biological variation, including the number and type of vessels present in a skin biopsy section.

In conclusion, we show that the NOTCH3 score in skin vessels is directly associated with stroke probability and neuroimaging outcomes in patients with CADASIL, and that the NOTCH3 score may contribute to genotype‐based risk classification for improved disease prediction.

## Author Contributions

M.N.C., G.G., J.W.R., and S.A.J.L.O. contributed to the conception and design of the study; M.N.C., G.G., R.J.H., K.L.D., M.R.G., B.G., M.N.W.W.‐A., R.vD., M.D., J.W.R., and S.A.J.L.O. contributed to the acquisition and analysis of data; M.N.C., G.G., J.W.R., and S.A.J.L.O. contributed to drafting the text or preparing the figures.

## Potential Conflicts of Interest

The authors declare no competing interests.

## Supporting information


**Data S1.** Supporting information.

## Data Availability

The data that support the findings of this study are available upon reasonable request from the corresponding author. The data are not publicly available due to privacy or ethical restrictions.
