# Supplementary material for: Association Between Vascular NOTCH3 Aggregation and Disease Severity in a CADASIL Cohort – Implications for NOTCH3 Variant‐Specific Disease Prediction
Source: Ann Neurol. 2025 Apr 23;98(2):273–85. doi: 10.1002/ana.27240 (PMC12278194; doi:10.1002/ana.27240)
Supplement: Supplementary file 1 — Data S1. Supporting information. [file ANA-98-273-s001.docx]

**SUPPLEMENTARY MATERIAL**

Supplementary Tables

**Supplementary Table 1. *NOTCH3*^cys^ variants of DiViNAS participants**

| **Nucleotide alteration** | **Protein alteration** | **Exon** | **EGFr domain** | ***NOTCH3* variant**  **risk category** | **Count** | **Percentage** |
| --- | --- | --- | --- | --- | --- | --- |
| c.160C>T | p.(Arg54Cys) | 2 | 1 | HR-*NOTCH3* | 3 | 1.4 |
| c.328C>T | p.(Arg110Cys) | 3 | 2 | HR-*NOTCH3* | 5 | 2.4 |
| c.350G>A | p.(Cys117Tyr) | 4 | 2 | HR-*NOTCH3* | 2 | 0.9 |
| c.397C>T | p.(Arg133Cys) | 4 | 3 | HR-*NOTCH3* | 3 | 1.4 |
| c.421C>T | p.(Arg141Cys) | 4 | 3 | HR-*NOTCH3* | 16 | 7.5 |
| c.431G>T | p.(Cys144Phe) | 4 | 3 | HR-*NOTCH3* | 4 | 1.9 |
| c.457C>T | p.(Arg153Cys) | 4 | 3 | HR-*NOTCH3* | 11 | 5.2 |
| c.486C>G | p.(Cys162Trp) | 4 | 4 | HR-*NOTCH3* | 2 | 0.9 |
| c.505C>T | p.(Arg169Cys) | 4 | 4 | HR-*NOTCH3* | 3 | 1.4 |
| c.544C>T | p.(Arg182Cys) | 4 | 4 | HR-*NOTCH3* | 12 | 5.7 |
| c.548G>A | p.(Cys183Tyr) | 4 | 4 | HR-*NOTCH3* | 2 | 0.9 |
| c.566A>G | p.(Tyr189Cys) | 4 | 4 | HR-*NOTCH3* | 1 | 0.5 |
| c.619C>T | p.(Arg207Cys) | 4 | 5 | HR-*NOTCH3* | 33 | 15.6 |
| c.634T>A | p.(Cys212Ser) | 4 | 5 | HR-*NOTCH3* | 1 | 0.5 |
| c.665G>A | p.(Cys222Tyr) | 4 | 5 | HR-*NOTCH3* | 1 | 0.5 |
| c.671G>A | p.(Cys224Tyr) | 4 | 5 | HR-*NOTCH3* | 1 | 0.5 |
| c.698G>A | p.(Cys233Tyr) | 5 | 5 | HR-*NOTCH3* | 1 | 0.5 |
| c.953G>T | p.(Cys318Phe) | 6 | 8 | HR-*NOTCH3* | 3 | 1.4 |
| c.1130G>A | p.(Cys377Tyr) | 7 | 9 | MR-*NOTCH3* | 2 | 0.9 |
| c.1187C>G | p.(Ser396Cys) | 7 | 10 | MR-*NOTCH3* | 2 | 0.9 |
| c.1241C>G | p.(Ser414Cys) | 8 | 10 | MR-*NOTCH3* | 1 | 0.5 |
| c.1261C>T | p.(Arg421Cys) | 8 | 10 | MR-*NOTCH3* | 1 | 0.5 |
| c.1279C>T | p.(Arg427Cys) | 8 | 10 | MR-*NOTCH3* | 1 | 0.5 |
| c.1345C>T | p.(Arg449Cys) | 8 | 11 | HR-*NOTCH3* | 2 | 0.9 |
| c.1591T>G | p.(Cys531Gly) | 10 | 13 | MR-*NOTCH3* | 2 | 0.9 |
| c.1630C>T | p.(Arg544Cys) | 11 | 13 - 14 | MR-*NOTCH3* | 3 | 1.4 |
| c.1672C>T | p.(Arg558Cys) | 11 | 14 | MR-*NOTCH3* | 3 | 1.4 |
| c.1703G>A | p.(Cys568Tyr) | 11 | 14 | MR-*NOTCH3* | 6 | 2.8 |
| c.1732C>T | p.(Arg578Cys) | 11 | 14 | MR-*NOTCH3* | 47 | 22.2 |
| c.1759C>T | p.(Arg587Cys) | 11 | 15 | MR-*NOTCH3* | 2 | 0.9 |
| c.1783G>T | p.(Gly595Cys) | 11 | 15 | MR-*NOTCH3* | 2 | 0.9 |
| c.1819C>T | p.(Arg607Cys) | 11 | 15 | MR-*NOTCH3* | 2 | 0.9 |
| c.1999G>T | p.(Gly667Cys) | 13 | 17 | MR-NOTCH3 | 9 | 4.2 |
| c.2182C>T | p.(Arg728Cys) | 14 | 18 | LR-*NOTCH3* | 4 | 1.9 |
| c.2984_2992del | p.(Pro995_Cys997del) | 18 | 25 | MR-*NOTCH3* | 1 | 0.5 |
| c.3043T>C | p.(Cys1015Arg) | 19 | 26 | HR-*NOTCH3* | 10 | 4.7 |
| c.3091C>T | p.(Arg1031Cys) | 19 | 26 | HR-*NOTCH3* | 1 | 0.5 |
| c.3226C>T | p.(Arg1076Cys) | 20 | 27 | MR-*NOTCH3* | 4 | 1.9 |
| c.3691C>T | p.(Arg1231Cys) | 22 | 31 | LR-*NOTCH3* | 3 | 1.4 |

**Abbreviations:** HR*-NOTCH3* = high-risk *NOTCH3* variant risk category; MR*-NOTCH3* = moderate-risk *NOTCH3* variant risk category; LR*-NOTCH3* = low-risk *NOTCH3* variant risk category

| **Patient** | **Protein alteration** | **EGFr domain** | ***NOTCH3* variant risk category** | **Sex** | **Age at time of death** | **Cause of death*** |
| --- | --- | --- | --- | --- | --- | --- |
| 1 | p.(Arg153Cys) | 3 | HR-*NOTCH3* | M | 60-65 | End-stage CADASIL |
| 2 | p.(Arg153Cys) | 3 | HR-*NOTCH3* | F | 55-60 | End-stage CADASIL |
| 3 | p.(Arg153Cys) | 3 | HR-*NOTCH3* | F | 55-60 | End-stage CADASIL |
| 4 | p.(Arg153Cys) | 3 | HR-*NOTCH3* | F | 55-60 | End-stage CADASIL |
| 5 | p.(Cys446Phe) | 11 | HR-*NOTCH3* | M | 65-70 | End-stage CADASIL |
| 6 | p.(Arg153Cys) | 3 | HR-*NOTCH3* | M | 65-70 | End-stage CADASIL |
| 7 | p.(Arg578Cys) | 14 | MR-*NOTCH3* | F | 80-85 | End-stage CADASIL |
| 8 | p.(Arg141Cys) | 3 | HR-*NOTCH3* | F | 55-60 | End-stage CADASIL |
| 9 | p.(Arg449Cys) | 11 | HR-*NOTCH3* | F | 60-65 | End-stage CADASIL |
| 10 | p.(Arg578Cys) | 14 | MR-*NOTCH3* | M | 70-75 | End-stage CADASIL |
| 11 | p.(Cys1015Arg) | 26 | HR-*NOTCH3* | F | 80-85 | End-stage CADASIL |
| 12 | p.(Arg578Cys) | 14 | MR-*NOTCH3* | M | 60-65 | End-stage CADASIL |

**Supplementary Table 2. Patient characteristics of brain donors**

*End-stage = bedridden with death due to selected complications such as dehydration, cachexia, and infection.**Supplementary Table 3. Baseline characteristics of 2-year follow-up cohort**

| Number of participants, n | 110 |
| --- | --- |
| *NOTCH3* variant risk category, n (%)   - HR-*NOTCH3* - MR-*NOTCH3* - LR-*NOTCH3* | 60 (54.5)  48 (43.6)  2 (1.8) |
| Age (years), mean (SD) | 54.5 (11.6) |
| Male sex, n (%) | 56 (50.9) |
| Ischemic stroke, n (%) | 35 (31.2) |
| Age of first stroke, mean (SD) | 52.7 (11.3) |
| Hypertension, n (%) | 27 (24.5) |
| Hypercholesterolemia, n (%) | 40 (36.4) |
| Diabetes type 1 or 2, n (%) | 7 (6.4) |
| Pack years, median (IQR) | 0 (7.95) |

**Abbreviations:** HR*-NOTCH3* = high-risk *NOTCH3* variant risk category; MR*-NOTCH3* = moderate-risk *NOTCH3* variant risk category; LR*-NOTCH3* = low-risk *NOTCH3* variant risk category; IQR = interquartile range

**Supplementary Table 4. Variant-specific NOTCH3 scores *vs* individual participants’ NOTCH3 scores**

| **Outcome** | **ΔAIC^a^** |
| --- | --- |
| PSMD | 32.3 |
| nWMHv | 51.0 |
| nLV | 42.4 |
| CMB count | 8 |
| PVS score | 10.5 |
| Lifetime stroke probability | 14.0 |

**Abbreviations:** AIC = Akaike information criterion; PSMD = peak width of the skeletonized mean diffusivity; nWMHv = normalized white matter hyperintensity volume; nLV = normalized lacune volume; CMB = cerebral microbleed; PVS = perivascular space.

a An ΔAIC of >2 indicates that the model with the variant-specific NOTCH3 score performed better than the model with individual participants’ NOTCH3 scores. **Supplementary Table 5. *Post-hoc* p.(Arg207Cys) comparisons**

| **Outcome^a^** | **HR-*NOTCH3* vs p.(Arg207Cys)** | **MR-*NOTCH3* vs p.(Arg207Cys)** |
| --- | --- | --- |
| The NOTCH3 score | *β*: 1.1  95% CI [0.78 – 1.4]  *p* = 4.9×10^-11^ | *β*: -0.079  95% CI [-0.37 – 0.21]  *p* = 0.60 |
| PSMD | *β*: 0.42  95% CI [0.17 – 0.68]  *p* = 1.6×10^-3^ | *β*: -0.52  95% CI [-0.77 to -0.27]  *p* = 7.2×10^-5^ |
| nWMHv | *β*: 0.60  95% CI [0.35 – 0.85]  *p* = 4.8×10^-6^ | *β*: -0.54  95% CI [-0.79 to -0.30]  *p* = 2.2×10^-5^ |
| nLV | *β*: 0.87  95% CI [0.54 – 1.20]  *p* = 1.1×10^-6^ | *β*: -0.14  95% CI [-0.47 – 0.19]  *p* = 0.42 |
| CMB count | *β*: 0.33  95% CI [-0.013 – 0.68]  *p* = 0.064 | *β*: -0.062  95% CI [-0.41 – 0.28]  *p* = 0.70 |
| Lifetime stroke probability | Hazard Ratio: 2.49  95% CI [1.3 – 4.77]  *p* = 0.0061 | Hazard Ratio: 0.46  95% CI [0.22 – 0.98]  *p* = 0.045 |

**Abbreviations:** PSMD = peak width of the skeletonized mean diffusivity; nWMHv = normalized white matter hyperintensity volume; nLV = normalized lacune volume; CMB = cerebral microbleed; PVS = perivascular space; BPF = brain parenchymal fraction.

a the p.(Arg207Cys) variant is used as a reference group in these comparisonsSupplementary Figures

**Supplementary Figure 1. The association between the NOTCH3 score and age, and sex**


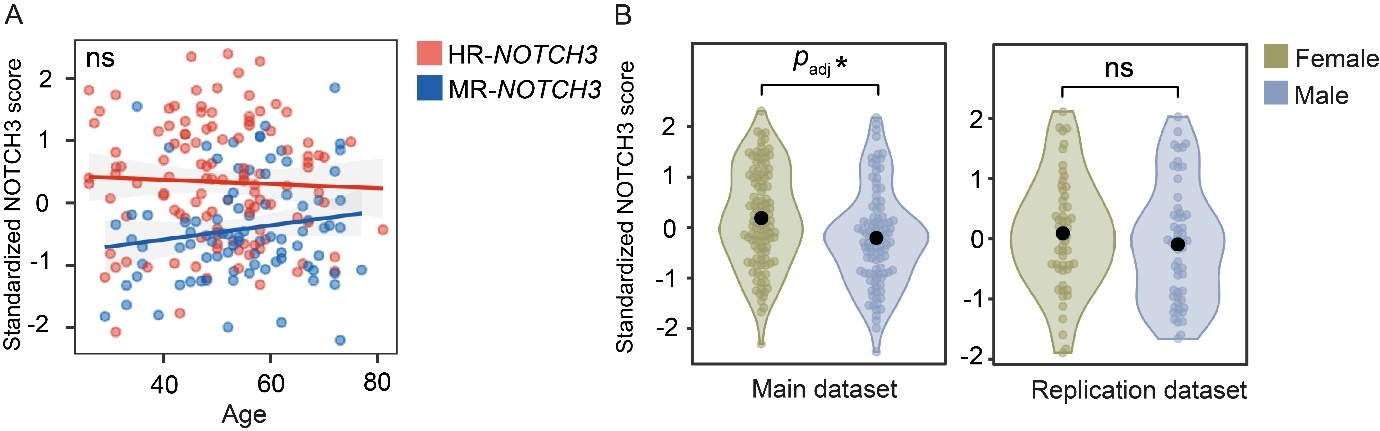


**Abbrevations:**; *p*_adj_ = multiple correction adjusted *p*-value (using the Holm method, n = 7); Levels of significance: **** = *p* < 0.0001, *** = *p* < 0.001, ** = p < 0.01, * = *p* < 0.05, ns = not significant.

The association between age and the NOTCH3 score did not differ between the HR- and MR-NOTCH3 groups (*p* = 0.14, A). There was a significant difference between males and females *(p* = 0.0019, *p*_adj_ = 0.011). In the replication dataset there was no difference between males and females (*p* = 0.36, B). **Supplementary Figure 2. Mediation analyses of the NOTCH3 score, *NOTCH3* variant risk category, and
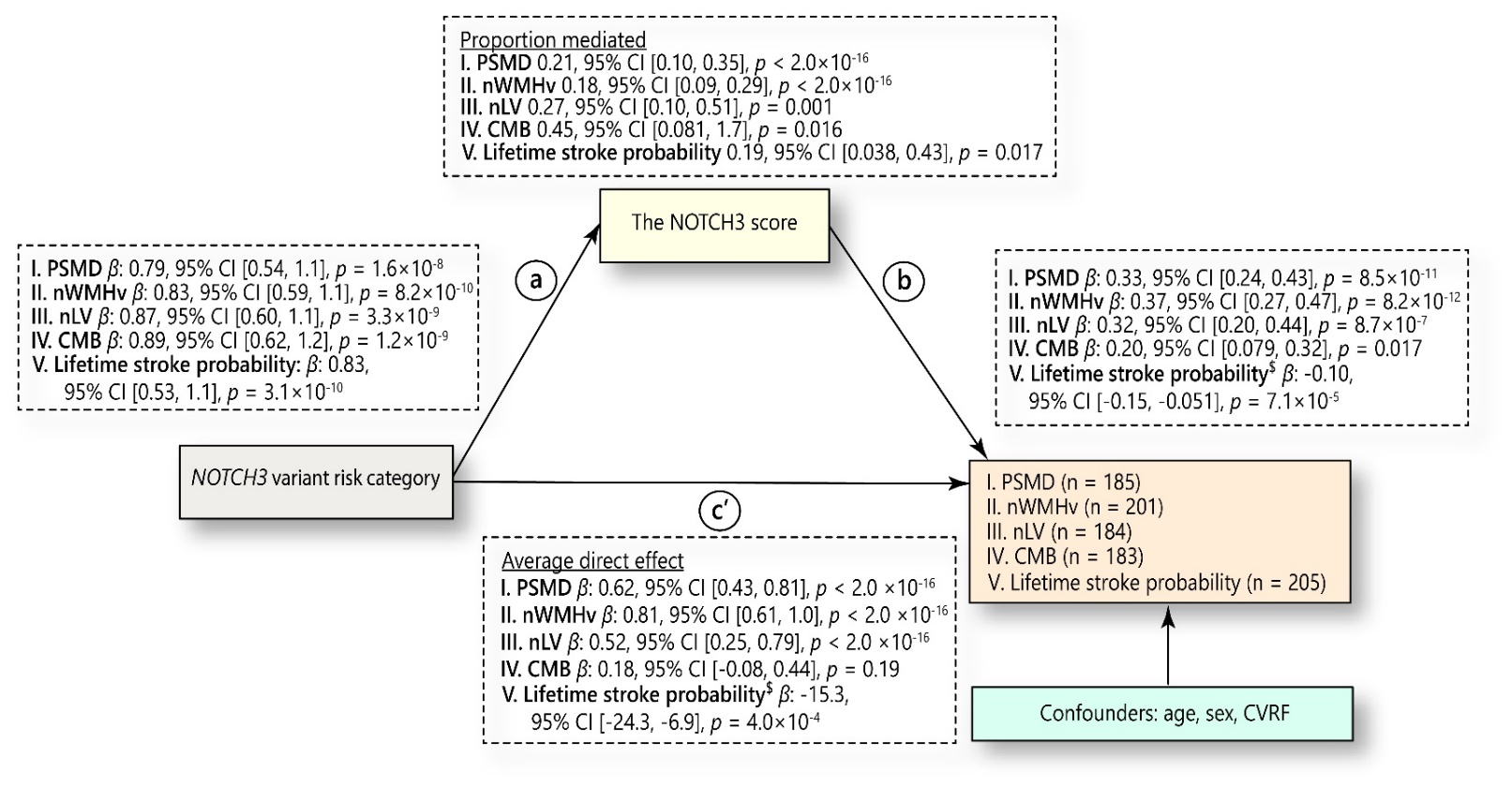
disease severity**

**Abbrevations:** CMB = cerebral microbleed; PSMD = peak width of the skeletonized mean diffusivity; nWMHv = normalized white matter hyperintensity volume; nLV = normalized lacune volume. Note that the number of participants (n) is different from the methods section, as only those with HR- and MR-*NOTCH3* variants were included. ^$^Coefficient *β* reported in paths b and *c’* for lifetime stroke probability is derived from a *parametric* survival model needed for mediation analysis, in which a negative value denotes a negative effect on survival time

Mediation path diagram of the effect of NOTCH3 aggregation on the association between *NOTCH3* variant risk category (HR- and MR-*NOTCH3* variants) and neuroimaging outcomes. The MR-NOTCH3 group is used as a reference for the reported estimates (*β*) for *NOTCH3* variant risk category. The association between *NOTCH3* variant risk category and PSMD (**I**), nWMHv (**II**), nLV (**III**), CMB count **(IV)** and lifetime stroke probability **(V)** was significantly mediated by the NOTCH3 score.

**Supplementary Figure 3. Plots of the average mediation effect as a function of the sensitivity parameter rho (mediation analysis)**


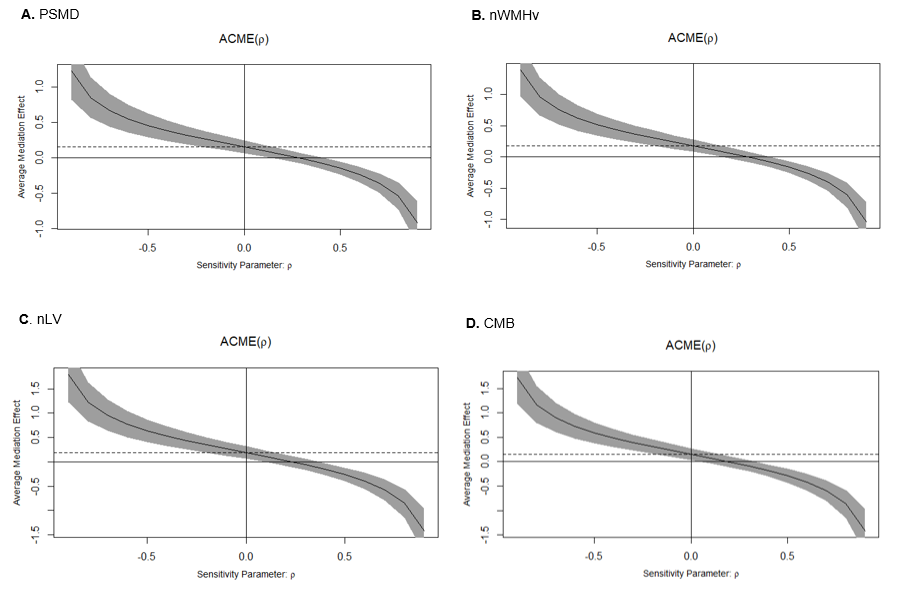


**Abbrevations**: CMB = cerebral microbleed; PSMD = peak width of the skeletonized mean diffusivity; nWMHv = normalized white matter hyperintensity volume; nLV = normalized lacune volume; ACME = average causal mediation effects.

Supplementary Results

When considering PVS score as an ordinal outcome, there was a significant association between NOTCH3 score and PVS score (*p* = 0.0025) and quartiles of PVS score (*p* = 0.0028). After stratification for *NOTCH3* variant risk category, these associations disappeared (*p* > 0.05 in both analyses).

In the analysis of the replication dataset, the NOTCH3 score was significantly associated with PSMD (n = 104, *β:* 0.48*,* 95% CI [0.35, 0.61], *p* = 2.6×10^-10^), nWMHv (n = 107, *β:* 0.48*,* 95% CI [0.34, 0.61], *p* = 1.9×10^-9^), nLV (n = 107, *β*: 0.42, 95% CI [0.26, 0.58], *p* = 3.2×10^-6^) and CMB count (n = 105, *β:* 0.23*,* 95% CI [0.057, 0.40], *p* = 0.011). After stratification for *NOTCH3* variant risk category, the association between the NOTCH3 score and PSMD (*β:* 0.32, 95% CI [0.19, 0.45], *p* = 1.6×10^-5^), nWMHv (*β*: 0.29, 95% CI [0.15, 0.42], *p* = 1.6×10^-4^) and nLV (*β*: 0.30, 95% CI [0.12, 0.47], *p* = 0.0020) remained significant, but not CMB count (*β*: 0.16, 95% CI [-0.034, 0.34], *p* = 0.1).
